# 7-(Pyrazol-4-yl)-3*H*-imidazo[4,5-*b*]pyridine-based derivatives for kinase inhibition: Co-crystallisation studies with Aurora-A reveal distinct differences in the orientation of the pyrazole *N*1-substituent

**DOI:** 10.1016/j.bmcl.2015.08.003

**Published:** 2015-10-01

**Authors:** Vassilios Bavetsias, Yolanda Pérez-Fuertes, Patrick J. McIntyre, Butrus Atrash, Magda Kosmopoulou, Lisa O’Fee, Rosemary Burke, Chongbo Sun, Amir Faisal, Katherine Bush, Sian Avery, Alan Henley, Florence I. Raynaud, Spiros Linardopoulos, Richard Bayliss, Julian Blagg

**Affiliations:** a Cancer Research UK Cancer Therapeutics Unit at The Institute of Cancer Research, London, United Kingdom; b University of Leicester, Department of Biochemistry, Lancaster Road, Leicester LE1 9HN, United Kingdom; c Division of Structural Biology, The Institute of Cancer Research, London, United Kingdom; d Breakthrough Breast Cancer Research Centre at The Institute of Cancer Research, London, United Kingdom

**Keywords:** Aurora-A, Imidazo[4,5-*b*]pyridine, Aurora kinase

## Abstract

Introduction of a 1-benzyl-1*H*-pyrazol-4-yl moiety at C7 of the imidazo[4,5-*b*]pyridine scaffold provided **7a** which inhibited a range of kinases including Aurora-A. Modification of the benzyl group in **7a**, and subsequent co-crystallisation of the resulting analogues with Aurora-A indicated distinct differences in binding mode dependent upon the pyrazole *N*-substituent. Compounds **7a** and **14d** interact with the P-loop whereas **14a** and **14b** engage with Thr217 in the post-hinge region. These crystallographic insights provide options for the design of compounds interacting with the DFG motif or with Thr217.

## Graphical abstract

**Figure:**
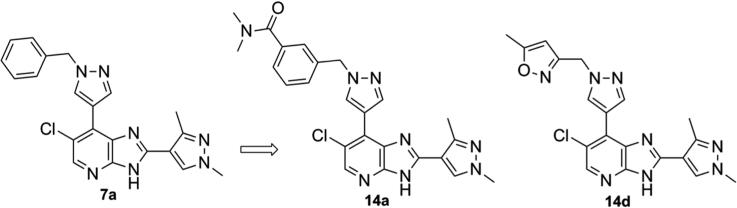

Over the last decade, extensive research has been directed towards the discovery of small molecule inhibitors of the Aurora protein kinases as anticancer agents. This effort led to the identification of multiple structurally diverse chemotypes for Aurora kinase modulation.^1–5^ In addition to small molecules inhibiting all three Aurora isoforms A, B and C, several compounds displaying Aurora isoform selectivity have also been reported including AZD1152 which selectively inhibits Aurora-B,^6^ and the selective Aurora-A inhibitor MLN8237.^7^ Several of these small-molecule inhibitors (e.g., PHA-739358,^8,9^ AZD1152 and MLN8237) have progressed through preclinical development into clinical evaluation for the treatment of a range of human malignancies.^1–5^

We have previously reported imidazo[4,5-*b*]pyridine-based inhibitors of Aurora kinases including **1** (CCT137690),^10^ the dual FLT3/Aurora kinase inhibitor **2** (CCT241736),^11^ and compound **3** which selectively inhibits Aurora-A over Aurora-B^12^ (Fig. 1). In our work related to the discovery of **3**, modification of the imidazo[4,5-*b*]pyridine scaffold at C7 included the introduction of a 1-benzyl-1*H*-pyrazol-4-yl moiety that provided entry into 7-(pyrazol-4-yl)-3*H*-imidazo[4,5-*b*]pyridine-based derivatives. Herein, we report our medicinal chemistry effort aimed at improving the pharmacological profile for this class of compounds, and present kinome profiling data that indicate promiscuous kinase inhibition for this subseries. In addition, we report ligand/Aurora-A protein crystallographic data that show different orientations for the substituent on the pyrazole ring suggesting that the 7-(pyrazol-4-yl)-3*H*-imidazo[4,5-*b*]pyridine scaffold could be utilised for the design of compounds that additionally interact with the DFG motif or with Thr217, the latter tactic has been demonstrated to enhance Aurora-A over Aurora-B selectivity.^12^

Synthesis of 7-substituted imidazo[4,5-*b*]pyridine derivatives **7a**–**e** (Table 1) is shown in Scheme 1. Key intermediates **5a**–**e** were obtained via Suzuki reaction between 4-chloro-3-nitropyridin-2-amine (**4**) and the requisite boronic acid or boronic acid pinacol ester coupling partner. 1-(3,4-Difluorobenzyl)-, 1-(4-fluorobenzyl)-, and 1-(4-chlorobenzyl)-1*H*-pyrazole-4-boronic acid pinacol esters were prepared by heating 4-(4,4,5,5-tetramethyl-1,3,2-dioxaborolan-2-yl)-1*H*-pyrazole (**11**) with the appropriate benzyl bromide at 80 °C for 2–3 h in acetonitrile in the presence of Cs_2_CO_3_, reaction conditions previously reported for the synthesis of 1-benzyl-3-heterocyclic pyrazoles.^13^ (1-Benzyl-1*H*-pyrazol-4-yl)boronic acid and (1-(4-fluorophenyl)-1*H*-pyrazol-4-yl)boronic acid, required for the synthesis of **5a** and **5e** respectively, were commercially available. *N*-chlorosuccinimide-mediated C5-pyridine chlorination was followed by reaction with 1,3-dimethyl-1*H*-pyrazole-4-carbaldehyde in the presence of Na_2_S_2_O_4_, as previously described,^11,12,14^ to afford the imidazo[4,5-*b*]pyridine derivatives **7a**–**e** (Scheme 1, Table 1).

The 2-amino-3-nitro-pyridine derivative **9** (Scheme 2), key intermediate for the synthesis of **14a** and **14b** (Table 2), was obtained from 4-chloro-3-nitropyridin-2-amine (**4**) by a Suzuki cross-coupling reaction to (1-(3-(methoxycarbonyl)benzyl)-1*H*-pyrazol-4-yl)boronic acid pinacol ester, prepared by reacting **11** with methyl 3-(bromomethyl)benzoate^13^, followed by C5-pyridine chlorination with *N*-chlorosuccinimide (Scheme 2). An attempt to prepare **10a** directly from the methyl ester intermediate **9** upon treatment with dimethylamine under microwave irradiation resulted in formation of the corresponding carboxylic acid. Coupling of this acid with dimethylamine via HATU carboxyl activation provided **10a** (Scheme 2). Starting from the methyl ester intermediate **9**, access to **10b** was achieved by alkaline ester hydrolysis followed by HATU-mediated coupling with 1-methylpiperazine (Scheme 2). Imidazo[4,5-*b*]pyridine ring formation to afford **14a** and **14b** (Table 2) was effected by reacting **10a** and **10b** with 1,3-dimethyl-1*H*-pyrazole-4-carbaldehyde in the presence of Na_2_S_2_O_4_ as previously described.^11,12,14^ The *ortho*-substituted derivative **14c** (Table 2) was prepared by a procedure analogous to that described for its *meta*-isomer **14b** (Scheme 2, Table 2).

Access to 2-amino-3-nitro-pyridine derivative **13** (Scheme 3), key intermediate for the preparation of **14e**–**g**, was achieved by alkylation of 4-(4,4,5,5-tetramethyl-1,3,2-dioxaborolan-2-yl)-1*H*-pyrazole (**11**) with 3-(bromomethyl)benzaldehyde^13^ followed by a Suzuki cross-coupling reaction with 4-chloro-3-nitropyridin-2-amine (**4**) (Scheme 3). Reductive amination of the benzaldehyde moiety in **13** was accomplished under standard conditions (NaBH(OAc)_3_/AcOH with methylamine or pyrrolidine, and NaBH_3_CN with dimethylamine). The synthesis of **14e**–**g** (Table 2) was finalised by the C5-pyridine chlorination of the requisite intermediate followed by imidazo[4,5-*b*]pyridine ring formation as described for the preparation of **7a**–**e** (Scheme 1). Finally, the isoxazole derivative **14d** (Table 2) was prepared from **4** by a route analogous to that described for the synthesis of **7a**–**e** (Scheme 1). For this synthetic sequence, (1-((5-methylisoxazol-3-yl)methyl)-1*H*-pyrazol-4-yl)boronic acid pinacol ester, required for the Suzuki cross-coupling reaction, was prepared by alkylation of 4-(4,4,5,5-tetramethyl-1,3,2-dioxaborolan-2-yl)-1*H*-pyrazole (**11**) with 3-(bromomethyl)-5-methylisoxazole.^13^

As part of our medicinal chemistry programme to discover selective inhibitors of Aurora-A,^12^ we introduced the 1-benzyl-1*H*-pyrazol-4-yl moiety at C7 of the imidazo[4,5-*b*]pyridine scaffold to provide **7a** (Table 1). We previously reported 1,3-dimethyl-1*H*-pyrazol-4-yl as a preferred substituent at the C2 position of the imidazo[4,5-*b*]pyridine scaffold^11^ and this moiety was therefore incorporated into the design of **7a** and analogues presented in this study. In biochemical assays, **7a** inhibited both Aurora-A and Aurora-B with IC_50_ values of 0.212 and 0.461 μM, respectively. A slightly improved level of target inhibition was observed in HeLa cervical cancer cells where **7a** inhibited the autophosphorylation of Aurora-A at T288 (a cell-based biomarker for Aurora-A inhibition)^12,15,16^ with an IC_50_ value of 0.087 μM and histone H3 phosphorylation at S10 (a cell-based biomarker for Aurora-B inhibition)^12,15,16^ with an IC_50_ value of 0.223 μM. Regarding cell growth inhibition, **7a** inhibited the growth of SW620 and HCT116 human colon carcinoma cells (GI_50_ = 0.18 and 0.15 μM, respectively).^19^ Compound **7a** displayed moderate mouse and human liver microsomal stability (40% and 43% metabolised, respectively, after a 30 min incubation). This in vitro profile prompted us to investigate this C7-pyrazole substituted imidazo[4,5-*b*]pyridine subseries in more detail. Firstly, we attempted to increase biochemical potency against Aurora-A, and also to improve microsomal stability.

Introduction of a *p*-F substituent in the phenyl ring of **7a** provided compound **7b** which displayed a similar Aurora-A/B inhibitory profile, and human liver microsomal stability to that of **7a**. No improvement in Aurora-A inhibitory potency was observed with the introduction of an additional fluorine (compound **7c**, Table 1), however the *p*-chlorobenzyl counterpart (compound **7d**, Table 1) displayed similar Aurora inhibitory activities to that of **7b** with improved human liver microsomal stability (16% metabolised after 30 min incubation with human liver microsomes). For a better understanding of the microsomal stability data, the protein binding for **7a**, **7d** and **7e** in microsomal incubations was measured. The percentage of **7a** unbound in MLM and HLM was determined as 6.71 and 4.89, respectively. Compounds **7d** and **7e** were more highly bound: for **7d** the percentage unbound was 0.50 in both MLM and HLM and for **7e** was 0.16 and 0.10, respectively. Based on these data, it is possible that the observed improvement in HLM metabolic stability for **7d** compared to **7a** is due to lower fraction unbound for the former; notably **7a** and **7d** display similar MLM stabilities despite the lower fraction unbound for **7d**. In HeLa cells, **7d** inhibited both Aurora-A (p-T288 IC_50_ = 0.040 μM) and Aurora-B (p-HH3 IC_50_ = 0.330 μM); potencies similar to those observed with compound **7a**. The direct attachment of a *p*-fluorophenyl group to the *N*1-pyrazole was detrimental to Aurora inhibition, with compound **7e** inhibiting Aurora-A and Aurora-B with IC_50_ values greater than 10 μM (Table 1). Cell growth inhibition studies revealed that compounds **7b**–**d** maintained the inhibitory potencies seen with **7a**; for example, **7d** inhibited the growth of SW620 and HCT116 cells (GI_50_ = 0.26 and 0.24 μM, respectively). Likewise, **7b** and **7c** showed potent HCT116 cell growth inhibition (GI_50_ = 0.20 and 0.13 μM, respectively). We postulated that potent cell growth inhibitory activity relative to biochemical Aurora-A and Aurora-B modulation may be attributable to gain of off-target kinase inhibition in this sub-series, although we acknowledge that translation to more potent cellular inhibition of Aurora-A versus Aurora-A biochemical potency may also be a contributing factor. The potent cell-based activity prompted us to investigate kinome selectivity profiles for this class of compound by screening **7a** and **7d** in a 102-kinase panel at a concentration of 1 μM.^20^ Indeed, both compounds inhibited a range of kinases including ERK8, GSK3β, MLK1, JAK2, TrkA and VEGFR greater than 80% (Table S1, Supplementary data) with Gini coefficients^21^ of 0.273 for **7a** and 0.364 for **7d**. These Gini coefficient values are significantly lower to those of previously reported 7-(piperazin-1-yl)-3*H*-imidazo[4,5-*b*]pyridine- and 7-phenoxy-3*H*-imidazo[4,5-*b*]pyridine-based inhibitors of Aurora kinases.^10,12^ The Gini coefficient for compound **3** was reported as 0.719,^12^ and the corresponding value for compound **1** is 0.560. To understand the potential for further improvement, we obtained the crystal structure of **7a** bound to Aurora-A^22^ (Fig. 3, Table S3, Supplementary data). This structure shows the ligand in the ATP binding site with the pyridine nitrogen atom hydrogen bonded to the backbone NH of Ala213 and the imidazole NH interacting with the carbonyl of Ala213, consistent with previous reports of the hinge binding mode for the imidazo[4,5-*b*]pyridine scaffold.^10–12^ Similar to crystal structures of previous compounds **1**, **15** and **16** (Figs. 1 and 2; compounds **51** and **40c** in Ref. 10, and compound **21a** in Ref. 11, respectively) the *N*-benzyl substituent on the C7-pyrazole is oriented towards the P-loop, making Van der Waals contacts with Val147 and Gly142 (Fig. 3).

In vivo mouse pharmacokinetic profiling of **7d** revealed low oral bioavailability (16%) with moderate clearance (0.016 L/h, 13.3 mL/min/kg) and volume of distribution (0.02 L, 1.0 L/kg). Similar pharmacokinetic parameters were observed for **7a** with low oral bioavailability (13%), moderate clearance (0.012 L/h, 10.0 mL/min/kg) and volume of distribution (0.01 L, 0.5 L/kg). The mouse plasma protein binding for **7a** and **7d** was determined as 99.48% and >99.9%, respectively. We determined low kinetic solubility for both **7a** and **7d** (<0.0001 mg/mL in phosphate buffer, pH = 6.8)^23^ which may be a contributing factor to the observed low oral bioavailability. In an attempt to improve the aqueous solubility, the Aurora inhibitory potency and kinase selectivity of this sub-series, we explored the introduction of basic substituents such as 1-methylpiperazine and pyrrolidine as well as replacement of the phenyl ring with a more polar heterocycle. This approach was guided by the ligand/protein interactions observed in the protein crystal structure of **7a**, which showed that the *N*-benzyl substituent on the C7-pyrazole is oriented towards the P-loop (Fig. 4). It was anticipated that the introduction of small substituents on the phenyl ring in **7a** would be well tolerated without altering its orientation in the kinase active site. Alternatively, we envisaged that Thr217 in Aurora-A may be accessed via an appropriate pyrazole *N*1-benzyl derivatisation such as a bulky amido substituent, which would form a favourable hydrogen bond interaction with the side chain hydroxyl of Thr217. Similar approaches were previously applied by us in the design of selective inhibitors of Aurora-A.^12,16^

Replacement of the phenyl ring in **7a** with 5-methylisoxazole (compound **14d**) led to a significant improvement in Aurora inhibition in our biochemical assays (Aurora-A IC_50_ = 0.035 μM, Aurora-B IC_50_ = 0.075 μM; Table 2) but promiscuous kinase inhibition remained. In a panel of 105 kinases at a compound concentration of 1 μM, 22 proteins including ERK8, MLK1, JAK2, TrkA and VEGFR were inhibited by ⩾80% (Gini coefficient = 0.320, Table S2, Supplementary data). Compound **14d** inhibited the growth of SW620 and HCT116 cells with GI_50_ values of 0.35 and 0.34 μM respectively, similar to the cell growth inhibition observed with **7d**. The crystal structure of **14d** bound to Aurora-A^22,24,25^ (Fig. 5, Table S3, Supplementary data) shows a similar binding mode to that adopted by **7a**; however, the P-loop of Aurora-A adopts a different conformation, stabilised by interactions with the 5-methylisoxazole; this P-loop conformation is very similar to that previously observed for compound **1** (compound **51** in Ref. 10).

The *m*-dimethylbenzamide derivative **14a** (Table 2) was a more potent inhibitor of Aurora-A compared to **7a** but its human liver microsomal stability was lower (72% metabolised after a 30 min incubation). A similar trend was observed with the *m*-1-methylpiperazine derivative **14b** which was also a more potent inhibitor of Aurora-A compared to **7a** but with poor metabolic stability in both mouse and human liver microsomes (Table 2). Both **14a** and **14b** were more potent (by 8- and 5-fold, respectively) inhibitors of Aurora-A relative to Aurora-B (Table 2). The crystal structures of **14a** and **14b** bound to Aurora-A^22,24,25^ were determined (Table S3, Supplementary data; Figs. 6 and 7) and revealed a consistent difference in the binding mode of these ligands when compared to **7a** and **14d**. Bulkier substituents on the pyrazole group no longer interact with the pocket formed by the two β-strands of the P-loop. Instead, these bulkier groups are positioned between the β1 strand of the P-loop and the post-hinge region. This orientation places them in close proximity to Thr 217, with a minimum interaction distance of 3.8 Å, which may explain the selectivity of these compounds towards Aurora-A. Notably, Aurora-B and Aurora-C have a glutamic acid at the equivalent position to Thr217, and computational modelling of this Thr to Glu replacement suggests that the bulkier R-groups of **14a** and **14b** may invoke a steric clash with a glutamic acid at position 217 (Fig. 6C, Fig. 7C).^12^

The *o*-regioisomer of **14b** (compound **14c**) displayed a similar profile to that of **14b** although its potency against Aurora-A was marginally lower and selectivity over Aurora-B was eroded (Table 2). The introduction of the smaller *m*-dimethylamino basic substituent (compound **14e**) led to potent inhibition of both Aurora-A and Aurora-B although mouse and human liver microsomal stability was poor (Table 2). Likewise, the *m*-pyrrolidine derivative **14f** potently inhibited both Aurora-A (IC_50_ = 0.017 μM) and Aurora-B (IC_50_ = 0.031 μM) but was also highly metabolised (Table 2). The potent Aurora-A and Aurora-B activity of these compounds suggests that they do not adopt a binding mode involving interaction with Thr217 in Aurora-A similar to **14a** and **14b**, but might bind similarly to **7a** and **14d**. Unfortunately, we have been unable to resolve the binding mode of compounds **14e** and **14f** by co-crystal structure determination to verify this hypothesis. The *m*-methylamino counterpart of **14e** (compound **14g**) also displayed potent inhibition of both Aurora-A (IC_50_ = 0.018 μM) and Aurora-B (IC_50_ = 0.032 μM) and demonstrated improved metabolic stability compared to **14e** and **14f** (Table 2). Compound **14g** inhibited the growth of SW620 and HCT116 cells (GI_50_ = 0.34 and 0.23 μM, respectively); however, profiling in a 105-kinase panel at a concentration of 1 μM revealed 30 kinases including ERK8, GSK3β, MLK1, JAK2, TrkA and VEGFR that were inhibited by ⩾80% (Gini coefficient = 0.267, Table S2, Supplementary data). Based on these findings, our interest in this 7-(pyrazol-4-yl)-3*H*-imidazo[4,5-*b*]pyridine-based sub-series was discontinued and our efforts focussed on compound **3** (Fig. 1) as a highly selective inhibitor of Aurora-A kinase.^12^ However, the kinase inhibitory profiles of compounds **7a**, **7d**, **14d**, and **14g** suggest that the 7-(pyrazol-4-yl)-3*H*-imidazo[4,5-*b*]pyridine scaffold could serve as the basis for a multitargeted kinase inhibitor design. In addition, the ligand/Aurora-A protein crystallographic data indicate distinct differences in the orientation of the pyrazole *N*-substituent. The phenyl group in **7a** and the 5-methylisoxazole in **14d** point towards the P-loop, whereas the bulkier benzamido substituted phenyl rings in **14a** and **14b** position between the β1 strand of the P-loop in close proximity to Thr217 and also to the DFG motif. These ligand/protein crystallographic insights could therefore be further exploited in the design of compounds interacting with the DFG motif or in enhancing the selectivity for Aurora-A inhibition over Aurora-B.

## Figures and Tables

**Figure 1:**
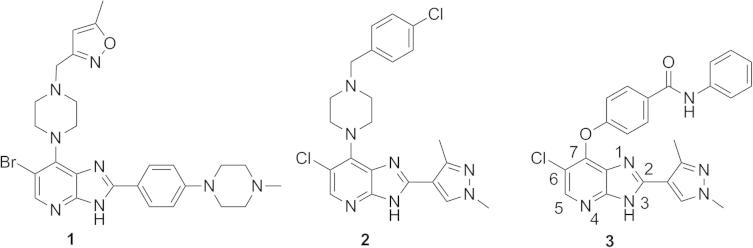
Imidazo[4,5-*b*]pyridine-based inhibitors of Aurora kinases.

**Figure 2:**
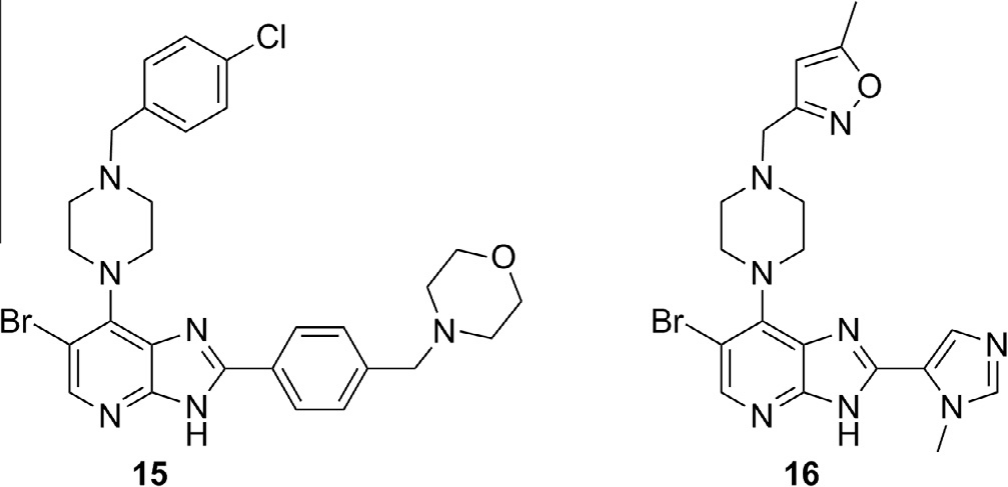
Imidazo[4,5-*b*]pyridine-based inhibitors of Aurora kinases co-crystallised with Aurora-A: compound **15**: PDB ID 2X6D;^10^ compound **16**: PDB ID 4B0G.^11^

**Figure 3:**
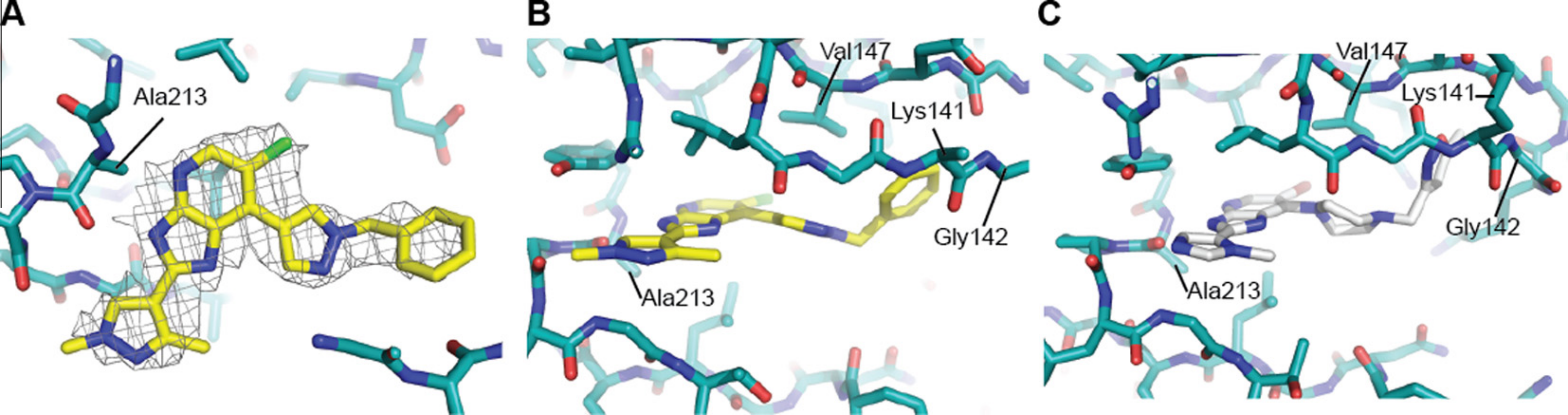
Crystal structure of **7a** bound to Aurora-A at 3.1 Å resolution. (A) The ATP binding site viewed from above. The P-loop has been removed to provide a clear view of the ligand, shown with carbon atoms coloured yellow, and the surrounding protein, shown with carbon atoms coloured teal. The final 2mFo-DFc electron density map is shown as a wire-mesh, contoured at 1 σ. (B) Side view with the P-loop shown and key interacting residues labelled. (C) Crystal structure of **16** (compound **21a** in Ref. 11; carbon atoms coloured white) bound to Aurora-A with the P-loop shown and key interacting residues labelled. Structure taken from the Protein Data Bank (PDB) ID 4B0G.

**Figure 4:**
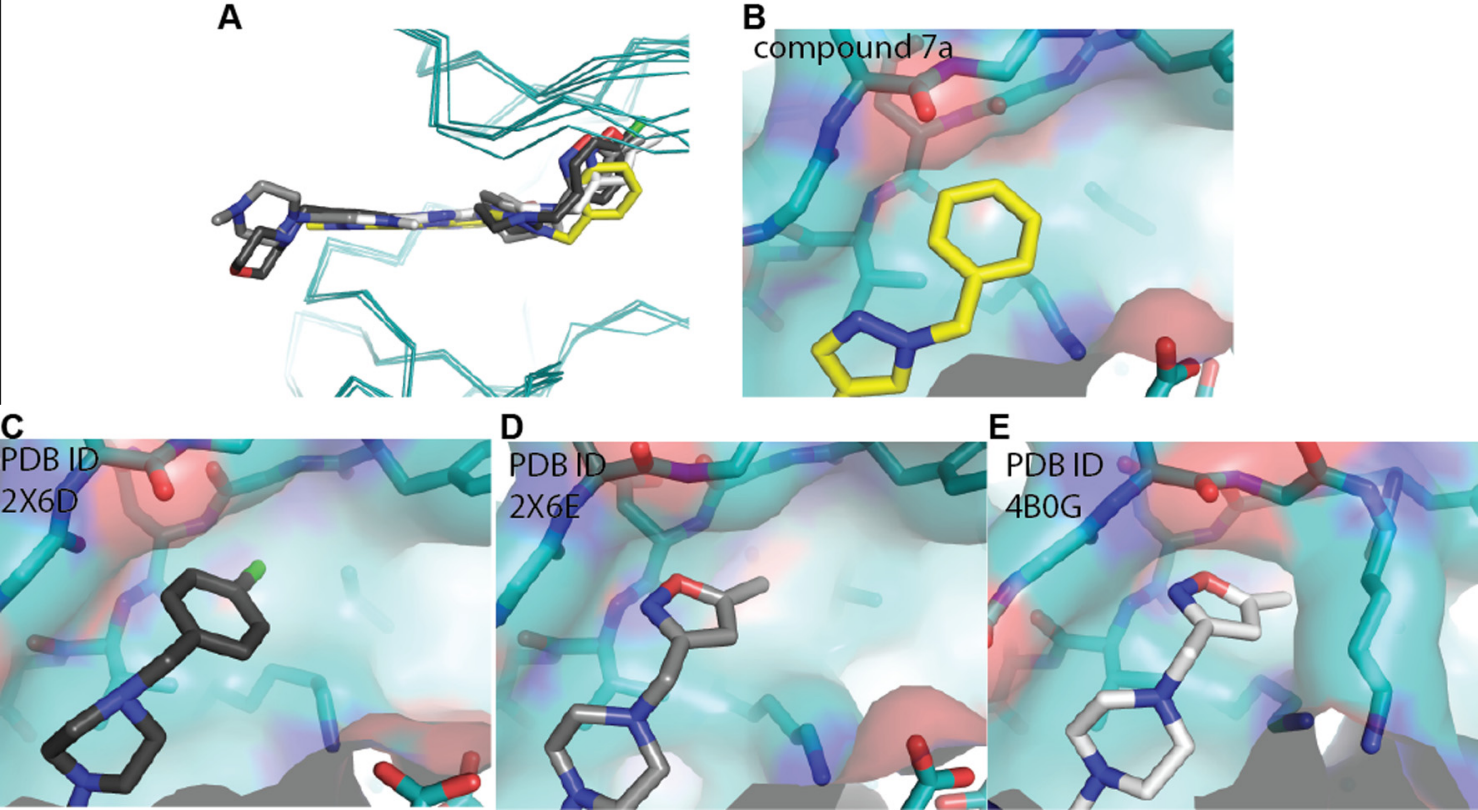
Comparison of crystal structure of Aurora-A bound to **7a** and published crystal structures from this series. (A) Overlay of structures showing superposed protein backbones (all coloured teal) with **7a** (carbon atoms coloured yellow), and published compounds **15**, **1** and **16** taken from PDB IDs 2X6D, 2X6E and 4B0G, respectively. (B–E) Magnified view of the interactions between compounds **7a**, **15**, **1** and **16** respectively and the P-loop with the protein surface shown.

**Figure 5:**
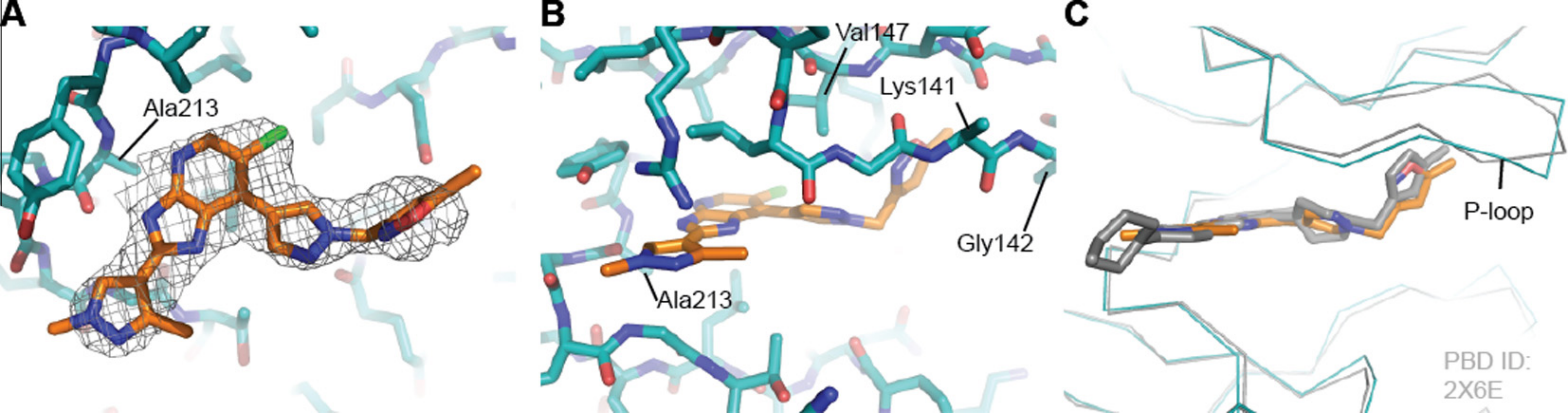
Crystal structure of **14d** bound to Aurora-A at 3.1 Å resolution. (A) The ATP binding site viewed from above. The P-loop has been removed to provide a clear view of the ligand, shown with carbon atoms coloured orange and the surrounding protein, shown with carbon atoms coloured teal. The final 2mFo-DFc electron density map is shown as a wire-mesh, contoured at 1 σ. (B) Side view with the P-loop shown and key interacting residues labelled. (C) Overlay of the structures of Aurora-A bound to compound **14d** (coloured as before) and compound **1** (compound and protein coloured grey).

**Figure 6:**
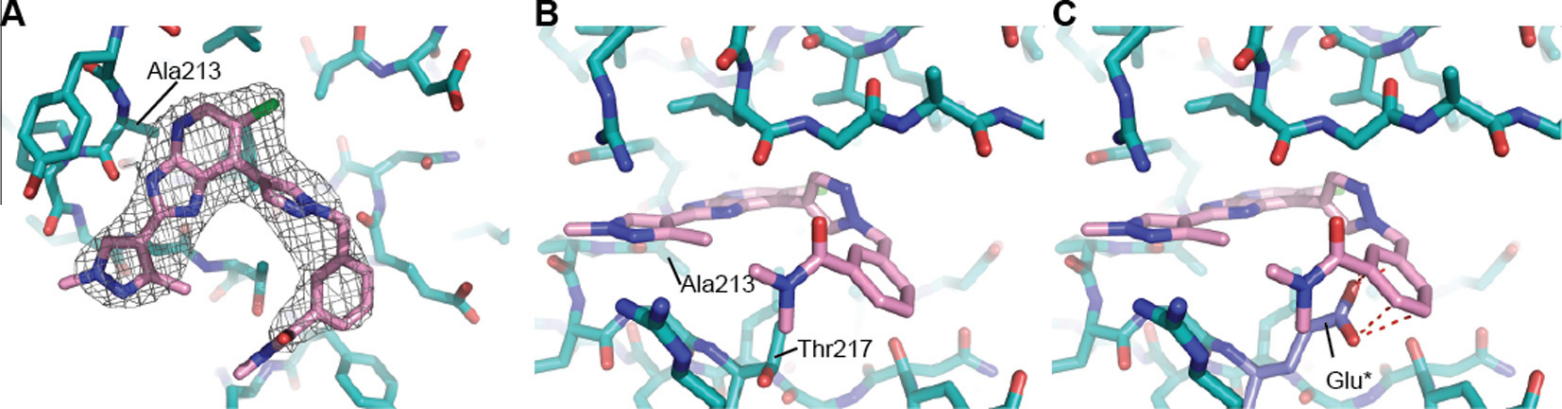
Crystal structure of **14a** bound to Aurora-A at 2.8 Å resolution. (A) The ATP binding site viewed from above. The P-loop has been removed to provide a clear view of the ligand, shown with carbon atoms coloured pink and the surrounding protein, shown with carbon atoms coloured teal. The final 2mFo-DFc electron density map is shown as a wire-mesh, contoured at 1 σ. (B) Side view with the P-loop shown and Ala213, Thr217 labelled. (C) Modelling a glutamic acid in position 217 (Glu^∗^), as found in Aurora-B, leads to steric clashes with the ligand, modelled clash shown as red dashes.

**Figure 7:**
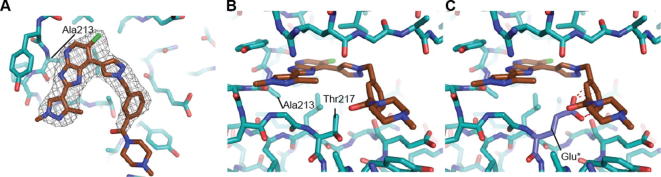
Crystal structure of **14b** bound to Aurora-A at 2.9 Å resolution. (A) The ATP binding site viewed from above. The P-loop has been removed to provide a clear view of the ligand, shown with carbon atoms coloured brown and the surrounding protein, shown with carbon atoms coloured teal. The final 2mFo-DFc electron density map is shown as a wire-mesh, contoured at 1 σ. (B) Side view with the P-loop shown and Ala213, Thr217 residues labelled. (C) Modelling a glutamic acid in position 217 (Glu^∗^), as found in Aurora-B, leads to steric clashes with the ligand, modelled clash shown as red dashes.

**Scheme 1:**
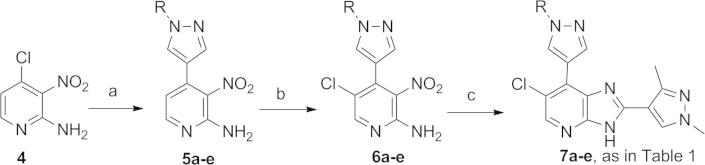
Reagents and conditions: (a) boronic acid or boronic acid pinacol ester, THF/H_2_O, Pd(dppf)Cl_2_, Na_2_CO_3_, 80 °C, 3.5–26 h or DME, Pd(dppf)Cl_2_, 1 M aqueous Na_2_CO_3_, 150 °C, microwave irradiation, 15 min (for the introduction of 1-(4-fluorophenyl)-1*H*-pyrazol-4-yl); (b) *N*-chlorosuccinimide, CH_3_CN, reflux, 3.5–7 h; (c) EtOH, 1,3-dimethyl-1*H*-pyrazole-4-carbaldehyde, 1 M aqueous Na_2_S_2_O_4_, 80 °C, 16–20 h.

**Scheme 2:**
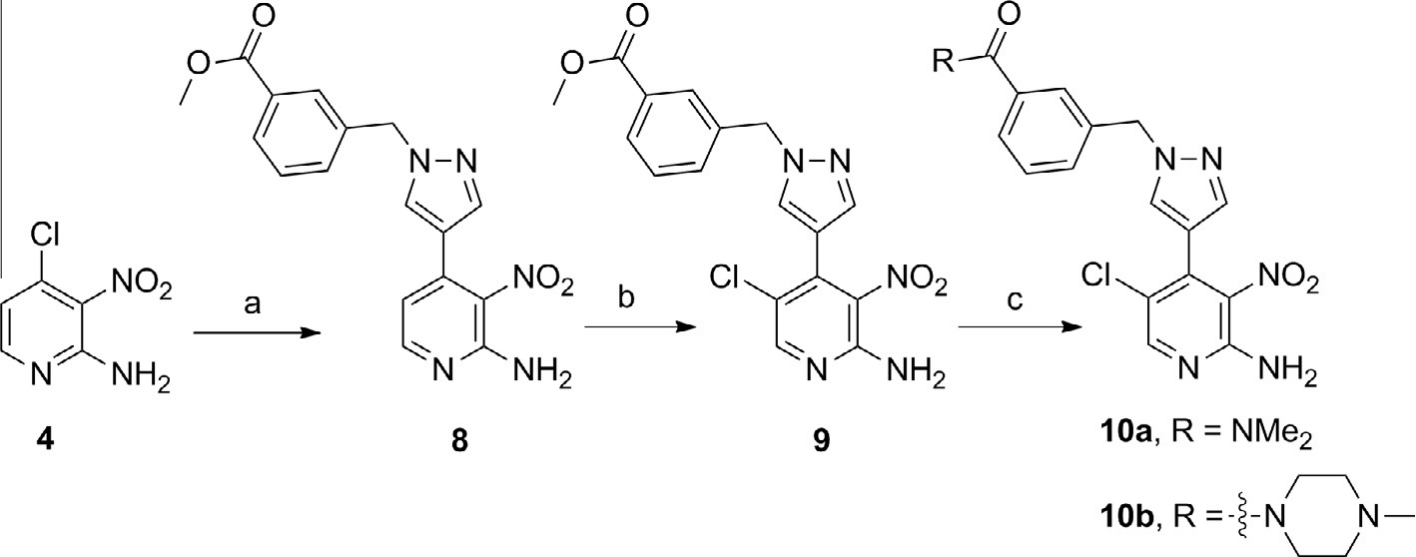
Reagents and conditions: (a) (1-(3-(methoxycarbonyl)benzyl)-1*H*-pyrazol-4-yl)boronic acid pinacol ester, DMF/H_2_O, Pd(dppf)Cl_2_, Na_2_CO_3_, 120 °C, microwave irradiation, 0.5 h; (b) *N*-chlorosuccinimide, CH_3_CN, reflux, 3.5 h; (c) for **10a**: (i) 2 M Me_2_NH in THF, 105 °C, microwave irradiation, 37 h then remove volatiles; (ii) DMF, HATU, *^i^*Pr_2_NEt, room temp, 1.7 h then 2 M Me_2_NH in THF, room temp, 2.3 h; for **10b**: (i) 1 M aqueous KOH, MeOH, 50 °C, 4.5 h; (ii) DMF, HATU, *^i^*Pr_2_NEt, room temp, 1 h then 1-methylpiperazine, room temp, 6 h.

**Scheme 3:**
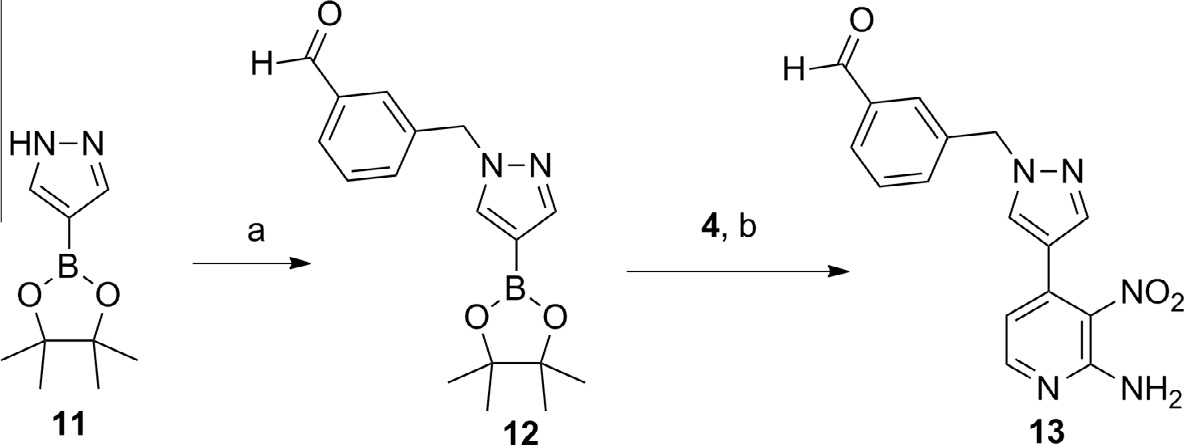
Reagents and conditions: (a) 3-(bromomethyl)benzaldehyde, CH_3_CN, Cs_2_CO_3_, 80 °C, 2 h; (b) THF/H_2_O, Pd(dppf)Cl_2_, Na_2_CO_3_, 80 °C, 2 h.

**Table 1:** C7-Pyrazole modifications

**Figure:**
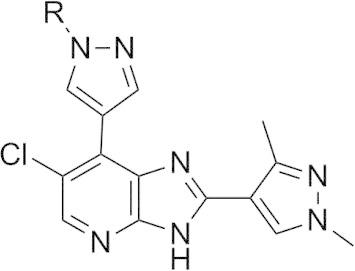

Compd	R	Aurora-A IC_50_ (μM)	Aurora-B IC_50_ (μM)	MLM/HLM% met/30 min
**7a**		0.212 ± 0.107	0.461 ± 0.147	40/43
**7b**		0.182	0.347	52/39
**7c**		0.322	0.458	31/28
**7d**		0.190	0.271	37/16
**7e**		>10	>10	31/20

IC_50_s are mean values of two independent determinations or mean (±SD) for *n* > 2.^17^

MLM/HLM: percentage of compound metabolised after a 30 min incubation.^18^

**Table 2:** Extended C7-pyrazole modifications

**Figure:**
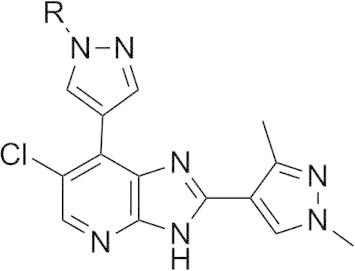

Compd	R	Aurora-A IC_50_ (μM)	Aurora-B IC_50_ (μM)	MLM/HLM% met/30 min
**14a**		0.036	0.296	72/72
**14b**		0.046	0.235	97/87
**14c**		0.126	0.354	98/82
**14d**		0.035	0.075	35/37
**14e**		0.014	0.017	97/95
**14f**		0.017	0.031	100/92
**14g**		0.018	0.032	53/38

IC_50_s are mean values of two independent determinations or mean (±SD) for *n* > 2.^17^

MLM/HLM: percentage of compound metabolised after a 30 min incubation.^18^

## References

[1] Pollard J.R., Mortimore M. (2009). J. Med. Chem..

[2] Green M.R., Woolery J.E., Mahadevan D. (2011). Expert Opin. Drug Discov..

[3] Cheung C.H.A., Coumar M.S., Chang J.-Y., Hsieh H.-P. (2011). Expert Opin. Ther. Patents.

[4] Kollareddy M., Zheleva D., Dzubak P., Brahmkshatriya P.S., Lepsik M., Hajduch M. (2012). Invest New Drugs.

[5] Gautschi O., Heighway J., Mack P.C., Purnell P.R., Lara P.N., Gandara D.R. (2008). Clin. Cancer Res..

[6] Mortlock A.A., Foote K.M., Heron N.M., Jung F.H., Pasquet G., Lohmann J.-J.M., Warin N., Renaud F., De Savi C., Roberts N.J., Johnson T., Dousson C.B., Hill G.B., Perkins D., Hatter G., Wilkinson R.W., Wedge S.R., Heaton S.P., Odedra R., Keen N.J., Crafter C., Brown E., Thompson K., Brightwell S., Khatri L., Brady M.C., Kearney S., McKillop D., Rhead S., Parry T., Green S. (2007). J. Med. Chem..

[7] Manfredi M.G., Ecsedy J.A., Chakravarty A., Silverman L., Zhang M., Hoar K.M., Stroud S.G., Chen W., Shinde V., Huck J.J., Wysong D.R., Janowick D.A., Hyer M.L., LeRoy P.J., Gershman R.E., Silva M.D., Germanos M.S., Bolen J.B., Claiborne C.F., Sells T.B. (2011). Clin. Cancer Res..

[8] Fancelli D., Moll J., Varasi M., Bravo R., Artico R., Berta D., Bindi S., Cameron A., Candiani I., Cappella P., Carpinelli P., Croci W., Forte B., Giorgini M.L., Klapwijk J., Marsiglio A., Pesenti E., Rocchetti M., Roletto F., Severino D., Soncini C., Storici P., Tonani R., Zugnoni P., Vianello P. (2006). J. Med. Chem..

[9] Caprinelli P., Ceruti R., Giorgini M.L., Cappella P., Gianellini L., Croci V., Degrassi A., Texido G., Rocchetti M., Vianello P., Rusconi L., Storici P., Zugnoni P., Arrigoni C., Soncini C., Alli C., Patton V., Marsiglio A., Ballinari D., Pesenti E., Fancelli D., Moll J. (2007). Mol. Cancer Ther..

[10] Bavetsias V., Large J.M., Sun C., Bouloc N., Kosmopoulou M., Matteucci M., Wilsher N.E., Martins V., Reynisson J., Atrash B., Faisal A., Urban F., Valenti M., de Haven Brandon A., Box G., Raynaud F.I., Workman P., Eccles S.A., Bayliss R., Blagg J., Linardopoulos S., McDonald E. (2010). J. Med. Chem..

[11] Bavetsias V., Crumpler S., Sun C., Avery S., Atrash B., Faisal A., Moore A.S., Kosmopoulou M., Brown N., Sheldrake P.W., Bush K., Henley A., Box G., Valenti M., de Haven Brandon A., Raynaud F.I., Workman P., Eccles S.A., Bayliss R., Linardopoulos S., Blagg J. (2012). J. Med. Chem..

[12] Bavetsias V., Faisal A., Crumpler S., Brown N., Kosmopoulou M., Joshi A., Atrash B., Pérez-Fuertes Y., Schmitt J.A., Boxall K.J., Burke R., Sun C., Avery S., Bush K., Henley A., Raynaud F.I., Workman P., Bayliss R., Linardopoulos S., Blagg J. (2013). J. Med Chem..

[13] Curtis M.P., Sammons M.F., Piotrowski D.W. (2009). Tetrahedron Lett..

[14] Yang D., Fokas D., Li J., Yu L., Baldino C.M. (2005). Synthesis.

[15] Manfredi M.G., Ecsedy J.A., Meetze K.A., Balani S.K., Burenkova O., Chen W., Galvin K.M., Hoar K.M., Huck J.J., LeRoy P.J., Ray E.T., Sells T.B., Stringer B., Stroud S.G., Vos T.J., Weatherhead G.S., Wysong D.R., Zhang M., Bolen J.B., Claiborne C.F. (2007). Proc. Natl. Acad. Sci. U.S.A..

[16] Bouloc N., Large J.M., Kosmopoulou M., Sun C., Faisal A., Matteucci M., Reynisson J., Brown N., Atrash B., Blagg J., McDonald E., Linardopoulos S., Bayliss R., Bavetsias V. (2010). Bioorg. Med. Chem. Lett..

[b0085] 17Aurora-A and Aurora-B IC_50_ values were determined by using a microfluidic assay which monitors the separation of a phosphorylated product from its substrate, as described in Ref. 12.

[b0090] 18MLM and HLM microsomal stabilities were determined as described in Ref. 12.

[19] Chan F., Sun C., Perumal M., Nguyen Q.-D., Bavetsias V., McDonald E., Martins V., Wilsher N., Raynaud F.I., Valenti M., Eccles S., te Poele R., Workman P., Aboagye E.O., Linardopoulos S. (2007). Mol. Cancer Ther..

[b0100] 20Kinase selectivity profiling for compounds **7a**, **7d**, **14d** and **14g** was carried out at the International Centre for Protein Kinase Profiling, Division of Signal Transduction Therapy, University of Dundee.

[21] Graczyk P.P. (2007). J. Med. Chem..

[b0110] 22The crystal structure of Aurora-A in complex with compound **7a** was determined as in Ref. 12. Other co-crystal structures used a mutant form of Aurora-A kinase domain (C290A, C393A), which has enhanced expression and generates superior crystals (Refs. ^24,25^). Hexagonal bipyramidal shaped crystals of Aurora A C290A,C393A were grown at 18 °C by hanging drop vapour diffusion in a 1 μL + 1 μL mixture of mother liquor (0.1 M Tris, pH 8.9; 0.2 M lithium sulfate; 30% w/v PEG 400) and Aurora A (12 mg/mL, 1 mM inhibitor compound, 5 mM MgCl_2_). Crystals were cryoprotected using 25% (v/v) ethylene glycol in mother liquor before cryo-cooling in liquid nitrogen. The structure was solved using molecular replacement with PDB:4CEG as the model and refinement was carried out as described in Ref. 12. Coordinates and structure factors have been deposited in the protein data bank with accession codes 5AAD, 5AAE, 5AAF, 5AAG (corresponding to the Aurora-A co-crystal structures of **7a**, **14d**, **14a** and **14b**, respectively).

[b0115] 23Solubility measurements were performed by Pharmorphix® Solid State Services, Member of the Sigma–Aldrich Group, Cambridge, UK.

[24] Rowan F.C., Richards M., Bibby R.A., Thompson A., Bayliss R., Blagg J. (2013). ACS Chem. Biol..

[25] Burgess S.G., Bayliss R. (2015). Acta Crystallogr. F Struct. Biol. Commun..

