# Supplementary material for: 7-(Pyrazol-4-yl)-3H-imidazo[4,5-b]pyridine-based derivatives for kinase inhibition: Co-crystallisation studies with Aurora-A reveal distinct differences in the orientation of the pyrazole N1-substituent
Source: Bioorg Med Chem Lett. 2015 Oct 1;25(19):4203–9. doi: 10.1016/j.bmcl.2015.08.003 (PMC4577729; doi:10.1016/j.bmcl.2015.08.003)
Supplement: Supplementary data — Supplementary Tables S1–S3. [file mmc1.docx]

**Supplementary Data**

**7-(Pyrazol-4-yl)-3*H*-imidazo[4,5-*b*]pyridine–based derivatives for kinase inhibition: Co-crystallisation studies with Aurora-A reveal distinct differences in the orientation of the pyrazole *N*1-substituent**

Vassilios Bavetsias,*^,a^ Yolanda Pérez-Fuertes,^a^ Patrick J. McIntyre,^b^ Butrus Atrash,^a^ Magda Kosmopoulou,^c^ Lisa O’Fee,^a^ Rosemary Burke,^a^ Chongbo Sun,^a^ Amir Faisal,^a,†^ Katherine Bush,^a^ Sian Avery,^a^ Alan Henley,^a^ Florence I. Raynaud,^a^ Spiros Linardopoulos,^a,d^ Richard Bayliss,*^,b,c^ Julian Blagg*^,a^

^a^Cancer Research UK Cancer Therapeutics Unit at The Institute of Cancer Research, London.

^b^University of Leicester, Department of Biochemistry, Lancaster Road, Leicester, LE1 9HN, United Kingdom.

^c^Division of Structural Biology, The Institute of Cancer Research, London.

^d^Breakthrough Breast Cancer Research Centre at The Institute of Cancer Research, London.

***Corresponding authors:** For V.B.: Telephone: +44 (0)20 87224158, E-mail: [vassilios.bavetsias@icr.ac.uk](mailto:vassilios.bavetsias@icr.ac.uk); For R.B.: Telephone: +44 (0)116 2297100, E-mail: [richard.bayliss@leicester.ac.uk](mailto:rb308@leicester.ac.uk) ; For J.B.: Telephone: +44(0)20 87224051, E-mail: [julian.blagg@icr.ac.uk](mailto:julian.blagg@icr.ac.uk)

^†^Current Address: SBA School of Science and Engineering, Lahore University of Management Sciences D.H.A. Lahore, Pakistan.

Pages 2-4: **Table S1**: Kinase selectivity profiling of compounds **7a** and **7d**.

Pages 5-8: **Table S2**: Kinase selectivity profiling of compounds **14d** and **14g**.

Page 9: **Table S3**: Summary of Crystallographic Analyses.

**Table S1:** Kinase selectivity profiling of compounds **7a** and **7d** in a 102-kinase panel screened at a concentration of 1 μM. The results are displayed as % activity remaining of assay duplicates with a standard deviation.

Compound **7a** compound **7d**

% activity %activity

Remaining SD remaining SD

| MKK1 | **28** | 7 | **27** | 4 |
| --- | --- | --- | --- | --- |
| ERK1 | **28** | 1 | **30** | 6 |
| ERK2 | **16** | 2 | **24** | 6 |
| JNK1 | **13** | 0 | **48** | 1 |
| JNK2 | **17** | 0 | **49** | 1 |
| JNK3 | **18** | 0 | **40** | 5 |
| p38a MAPK | **90** | 5 | **91** | 1 |
| p38b MAPK | **91** | 15 | **100** | 14 |
| p38g MAPK | **17** | 2 | **55** | 6 |
| p38d MAPK | **18** | 0 | **52** | 3 |
| ERK8 | **6** | 0 | **8** | 0 |
| RSK1 | **25** | 0 | **26** | 1 |
| RSK2 | **42** | 4 | **43** | 2 |
| PDK1 | **52** | 3 | **87** | 1 |
| PKBa | **70** | 6 | **74** | 4 |
| PKBb | **62** | 1 | **105** | 21 |
| SGK1 | **56** | 4 | **83** | 3 |
| S6K1 | **18** | 1 | **37** | 2 |
| PKA | **72** | 10 | **62** | 3 |
| ROCK 2 | **36** | 4 | **57** | 3 |
| PRK2 | **42** | 3 | **57** | 11 |
| PKCa | **63** | 1 | **57** | 1 |
| PKCz | **103** | 20 | **86** | 1 |
| PKD1 | **76** | 8 | **92** | 11 |
| MSK1 | **62** | 4 | **59** | 8 |
| MNK1 | **55** | 1 | **27** | 8 |
| MNK2 | **44** | 2 | **35** | 0 |
| MAPKAP-K2 | **84** | 4 | **102** | 3 |
| MAPKAP-K3 | **69** | 8 | **79** | 9 |
| PRAK | **83** | 5 | **89** | 10 |
| CAMKKb | **19** | 1 | **32** | 2 |
| CAMK1 | **87** | 7 | **101** | 10 |
| SmMLCK | **51** | 1 | **49** | 4 |
| PHK | **17** | 3 | **68** | 5 |
| CHK1 | **68** | 0 | **74** | 1 |
| CHK2 | **26** | 2 | **38** | 3 |
| GSK3b | **2** | 0 | **3** | 0 |
| CDK2-Cyclin A | **1** | 0 | **3** | 1 |
| PLK1 | **75** | 6 | **70** | 2 |
| Aurora A | **1** | 0 | **1** | 0 |
| Aurora B | **74** | 20 | **114** | 30 |
| LKB1 | **49** | 2 | **64** | 3 |
| AMPK | **50** | 5 | **59** | 5 |
| MARK1 | **31** | 4 | **51** | 7 |
| MARK2 | **31** | 5 | **42** | 0 |
| MARK3 | **20** | 3 | **25** | 0 |
| MARK4 | **37** | 1 | **60** | 2 |
| BRSK1 | **36** | 4 | **35** | 3 |
| BRSK2 | **33** | 5 | **33** | 7 |
| MELK | **19** | 3 | **27** | 3 |
| NUAK1 | **1** | 0 | **4** | 0 |
| CK1 | **63** | 9 | **92** | 7 |
| CK2 | **62** | 12 | **30** | 7 |
| DYRK1A | **3** | 0 | **8** | 0 |
| DYRK2 | **29** | 2 | **15** | 2 |
| DYRK3 | **25** | 2 | **19** | 2 |
| NEK2a | **67** | 4 | **68** | 1 |
| NEK6 | **64** | 11 | **77** | 13 |
| IKKb | **48** | 7 | **68** | 3 |
| IKKe | **53** | 40 | **32** | 3 |
| TBK1 | **24** | 3 | **46** | 6 |
| PIM1 | **71** | 4 | **91** | 3 |
| PIM2 | **86** | 18 | **98** | 8 |
| PIM3 | **45** | 1 | **80** | 1 |
| SRPK1 | **73** | 10 | **72** | 3 |
| EF2K | **94** | 4 | **101** | 9 |
| HIPK1 | **3** | 1 | **5** | 1 |
| HIPK2 | **6** | 1 | **6** | 0 |
| HIPK3 | **9** | 1 | **9** | 1 |
| PAK2 | **82** | 15 | **103** | 25 |
| PAK4 | **23** | 0 | **38** | 1 |
| PAK5 | **36** | 1 | **49** | 6 |
| PAK6 | **42** | 1 | **48** | 0 |
| MST2 | **60** | 10 | **77** | 2 |
| MST4 | **64** | 22 | **58** | 14 |
| GCK | **22** | 2 | **23** | 1 |
| MINK1 | **28** | 2 | **44** | 9 |
| MEKK1 | **74** | 4 | **78** | 21 |
| MLK1 | **3** | 0 | **6** | 1 |
| MLK3 | **2** | 0 | **3** | 1 |
| TAK1 | **6** | 0 | **16** | 1 |
| IRAK4 | **28** | 0 | **52** | 5 |
| RIPK2 | **40** | 11 | **59** | 0 |
| TTK | **29** | 2 | **37** | 1 |
| Src | **9** | 1 | **32** | 4 |
| Lck | **61** | 15 | **88** | 8 |
| CSK | **59** | 7 | **80** | 2 |
| YES1 | **12** | 2 | **39** | 10 |
| BTK | **46** | 2 | **76** | 2 |
| JAK2 | **2** | 0 | **4** | 1 |
| SYK | **37** | 0 | **74** | 21 |
| EPH-A2 | **70** | 2 | **91** | 4 |
| EPH-A4 | **54** | 4 | **90** | 3 |
| EPH-B3 | **99** | 11 | **86** | 1 |
| EPH-B4 | **62** | 3 | **94** | 9 |
| FGF-R1 | **5** | 1 | **13** | 4 |
| HER4 | **89** | 20 | **104** | 14 |
| IGF-1R | **10** | 2 | **8** | 0 |
| IR | **3** | 0 | **34** | 14 |
| IRR | **20** | 1 | **26** | 2 |
| TrkA | **6** | 4 | **8** | 2 |
| VEG-FR | **6** | 1 | **7** | 0 |

**Table S2:** Kinase selectivity profiling of compounds **14d** and **14g** in a 105-kinase panel screened at a concentration of 1 μM. The results are displayed as % activity remaining of assay duplicates with a standard deviation.

Compound **14d** compound **14g**

% activity %activity

remaining SD remaining SD

|  |  | |  | |
| --- | --- | --- | --- | --- |
| MKK1 | **40** | 5 | **39** | 4 |
| ERK1 | **52** | 3 | **38** | 4 |
| ERK2 | **32** | 2 | **18** | 6 |
| JNK1 | **45** | 3 | **26** | 2 |
| JNK2 | **48** | 3 | **35** | 1 |
| JNK3 | **62** | 25 | **40** | 13 |
| p38a MAPK | **85** | 14 | **94** | 2 |
| p38b MAPK | **91** | 1 | **74** | 13 |
| p38g MAPK | **36** | 1 | **48** | 1 |
| p38d MAPK | **41** | 6 | **37** | 4 |
| ERK8 | **1** | 0 | **1** | 0 |
| RSK1 | **28** | 3 | **20** | 1 |
| RSK2 | **20** | 2 | **10** | 2 |
| PDK1 | **49** | 1 | **27** | 1 |
| PKBa | **99** | 9 | **84** | 5 |
| PKBb | **94** | 2 | **70** | 14 |
| SGK1 | **65** | 0 | **25** | 3 |
| S6K1 | **12** | 3 | **13** | 3 |
| PKA | **63** | 1 | **42** | 4 |
| ROCK 2 | **44** | 1 | **32** | 1 |
| PRK2 | **43** | 9 | **30** | 4 |
| PKCa | **83** | 1 | **45** | 2 |
| PKCz | **96** | 9 | **82** | 5 |
| PKD1 | **97** | 5 | **82** | 1 |
| MSK1 | **61** | 13 | **50** | 9 |
| MNK1 | **64** | 7 | **62** | 8 |
| MNK2 | **32** | 1 | **51** | 0 |
| MAPKAP-K2 | **100** | 3 | **84** | 1 |
| MAPKAP-K3 | **103** | 3 | **95** | 2 |
| PRAK | **100** | 5 | **110** | 7 |
| CAMKKb | **42** | 2 | **51** | 6 |
| CAMK1 | **77** | 5 | **43** | 1 |
| SmMLCK | **43** | 55 | **68** | 1 |
| PHK | **27** | 0 | **19** | 3 |
| DAPK1 | **24** | 8 | **12** | 2 |
| CHK1 | **52** | 9 | **59** | 10 |
| CHK2 | **32** | 0 | **25** | 1 |
| GSK3b | **47** | 65 | **2** | 0 |
| CDK2-Cyclin A | **1** | 0 | **2** | 0 |
| PLK1 | **115** | 5 | **101** | 8 |
| Aurora A | **1** | 0 | **1** | 0 |
| Aurora B | **8** | 1 | **8** | 0 |
| LKB1 | **62** | 10 | **66** | 8 |
| AMPK | **34** | 5 | **18** | 5 |
| MARK1 | **38** | 0 | **56** | 3 |
| MARK2 | **28** | 1 | **48** | 4 |
| MARK3 | **19** | 1 | **44** | 1 |
| MARK4 | **25** | 2 | **33** | 5 |
| BRSK1 | **61** | 10 | **56** | 8 |
| BRSK2 | **72** | 1 | **77** | 5 |
| MELK | **49** | 3 | **58** | 52 |
| NUAK1 | **8** | 0 | **8** | 0 |
| CK1 | **78** | 8 | **49** | 5 |
| CK2 | **57** | 3 | **55** | 8 |
| DYRK1A | **9** | 0 | **14** | 1 |
| DYRK2 | **34** | 4 | **58** | 3 |
| DYRK3 | **30** | 2 | **36** | 2 |
| NEK2a | **71** | 4 | **86** | 2 |
| NEK6 | **116** | 4 | **80** | 5 |
| IKKb | **63** | 2 | **69** | 2 |
| IKKe | **3** | 1 | **10** | 1 |
| TBK1 | **7** | 0 | **19** | 0 |
| PIM1 | **96** | 37 | **53** | 4 |
| PIM2 | **91** | 8 | **80** | 6 |
| PIM3 | **55** | 6 | **34** | 1 |
| SRPK1 | **31** | 36 | **53** | 2 |
| EF2K | **102** | 1 | **92** | 9 |
| HIPK1 | **22** | 1 | **21** | 1 |
| HIPK2 | **11** | 1 | **10** | 0 |
| HIPK3 | **49** | 3 | **57** | 5 |
| CLK2 | **2** | 0 | **3** | 0 |
| PAK2 | **102** | 8 | **80** | 7 |
| PAK4 | **25** | 4 | **51** | 1 |
| PAK5 | **41** | 0 | **48** | 2 |
| PAK6 | **45** | 1 | **65** | 2 |
| MST2 | **80** | 12 | **80** | 3 |
| MST4 | **56** | 3 | **50** | 2 |
| GCK | **40** | 2 | **51** | 1 |
| MINK1 | **86** | 35 | **56** | 1 |
| MEKK1 | **66** | 58 | **12** | 1 |
| MLK1 | **8** | 2 | **8** | 1 |
| MLK3 | **6** | 2 | **4** | 1 |
| TAK1 | **18** | 1 | **24** | 2 |
| IRAK4 | **33** | 1 | **47** | 0 |
| RIPK2 | **49** | 0 | **68** | 4 |
| TTK | **42** | 2 | **45** | 2 |
| Src | **8** | 1 | **4** | 0 |
| Lck | **34** | 1 | **17** | 1 |
| CSK | **83** | 2 | **62** | 1 |
| YES1 | **18** | 2 | **8** | 3 |
| BTK | **33** | 0 | **13** | 0 |
| JAK2 | **2** | 0 | **4** | 0 |
| SYK | **49** | 0 | **30** | 1 |
| EPH-A2 | **57** | 0 | **47** | 0 |
| EPH-A4 | **51** | 4 | **29** | 4 |
| EPH-B2 | **60** | 2 | **70** | 9 |
| EPH-B3 | **54** | 1 | **39** | 3 |
| EPH-B4 | **86** | 5 | **74** | 6 |
| FGF-R1 | **7** | 3 | **7** | 3 |
| HER4 | **73** | 3 | **43** | 2 |
| IGF-1R | **5** | 0 | **3** | 1 |
| IR | **53** | 3 | **20** | 0 |
| IRR | **24** | 0 | **23** | 3 |
| TrkA | **15** | 4 | **8** | 1 |
| VEG-FR | **7** | 2 | **5** | 2 |

**Table S3**: Summary of Crystallographic Analyses

|  | Compound **7a** | Compound **14d** | Compound **14b** | Compound **14a** |
| --- | --- | --- | --- | --- |
| *PDB code* | 5AAD | 5AAE | 5AAG | 5AAF |
|  |  |  |  |  |
| *Lattice parameters* |  |  |  |  |
| Space group | *P*6_1_22 | *P*6_1_22 | *P*6_1_22 | *P*6_1_22 |
| *a* (Å) | 81.14 | 81.78 | 82.59 | 82.51 |
| *b* (Å) | 81.14 | 81.78 | 82.59 | 82.51 |
| *c* (Å) | 171.53 | 167.84 | 168.84 | 169.95 |
| α (°) | 90.00 | 90.00 | 90.00 | 90.00 |
| β (°) | 90.00 | 90.00 | 90.00 | 90.00 |
| γ (°) | 120.00 | 120.00 | 120.00 | 120.00 |
|  |  |  |  |  |
| *Data collection* |  |  |  |  |
| Diamond beamline | I04 | I03 | I03 | I03 |
| Wavelength (Å) | 0.9789 | 0.9763 | 0.9763 | 0.9763 |
| Resolution range | 85.76-3.10 | 54.12 – 3.11 | 65.86 – 2.85 | 65.85 – 2.78 |
| (highest-resolution shell) (Å) | 3.27-3.10 | 3.19 – 3.11 | 2.92 – 2.85 | 2.85 – 2.78 |
| Unique reflections | 6489 (924) | 6482 (457) | 8526 (604) | 9168 (662) |
| Completeness (%) | 98.8 (99.6) | 99.8 (99.6) | 99.9 (100.0) | 99.9 (100.0) |
| Multiplicity | 7.6 (8.0) | 8.7 (9.5) | 9.0 (9.5) | 9.2 (9.8) |
| *R*_merge_ (%) | 8.4 (45.4) | 5.0 (81.4) | 3.9 (59.2) | 5.0 (54.9) |
| *I*/σ(*I*) | 16.3 (4.4) | 23.4 (2.6) | 28.9 (3.7) | 23.0 (3.7) |
|  |  |  |  |  |
|  |  |  |  |  |
| Refinement |  |  |  |  |
| Resolution range (Å) | 54.36 – 3.10 | 54.13 – 3.11 | 54.58 – 2.85 | 54.66 – 2.78 |
| Rfactor (%) | 20.10 | 24.41 | 21.34 | 23.97 |
| Rfree (%) | 28.27 | 28.01 | 26.02 | 28.71 |
| Bond deviation (Å) | 0.011 | 0.005 | 0.002 | 0.004 |
| Angle deviation (°) | 1.264 | 1.090 | 0.503 | 0.804 |
|  |  |  |  |  |
| MolProbity analysis |  |  |  |  |
| All-atom Clashscore | 16.97 | 14.89 | 4.92 | 6.78 |
| Rotamer outliers (%) | 6.4 | 2.4 | 0.0 | 1.0 |
| Ramachandran outliers (%) | 0.8 | 2.0 | 0.4 | 0.4 |
| Ramachandran favoured (%) | 90.7 | 91.6 | 95.6 | 95.2 |
